# P-value based visualization of codon usage data

**DOI:** 10.1186/1748-7188-1-10

**Published:** 2006-06-29

**Authors:** Peter Meinicke, Thomas Brodag, Wolfgang Florian Fricke, Stephan Waack

**Affiliations:** 1 Abteilung Bioinformatik, Institut für Mikrobiologie und Genetik, Georg-August-Universität Göttingen, Goldschmidtstr. 1, 37077 Göttingen, Germany; 2 Institut für Numerische und Angewandte Mathematik, Universität Göttingen, Lotzestr. 16, 37083 Göttingen, Germany; 3 Göttingen Genomics Laboratory, Universität Göttingen, Grisebachstr. 8, 37077 Göttingen, Germany

## Abstract

Two important and not yet solved problems in bacterial genome research are the identification of horizontally transferred genes and the prediction of gene expression levels. Both problems can be addressed by multivariate analysis of codon usage data. In particular dimensionality reduction methods for visualization of multivariate data have shown to be effective tools for codon usage analysis. We here propose a multidimensional scaling approach using a novel similarity measure for codon usage tables. Our probabilistic similarity measure is based on P-values derived from the well-known chi-square test for comparison of two distributions. Experimental results on four microbial genomes indicate that the new method is well-suited for the analysis of horizontal gene transfer and translational selection. As compared with the widely-used correspondence analysis, our method did not suffer from outlier sensitivity and showed a better clustering of putative alien genes in most cases.

## Background

The standard genetic code of protein coding DNA sequences shows a redundancy, since different triplet codons may be used to code for the same amino acid. In general, codon usages show organism-specific patterns. However, codon usage variation within a single genome can be an important source of information about gene expression levels and events of horizontal gene transfer. In particular, dimensionality reduction methods have widely been used for the analysis of codon usage patterns in microbial genomes. These methods provide a low-dimensional point representation of genes, where the proximity of gene-specific points indicates a similar codon usage of the associated genes. Hence, the resulting two-dimensional scatter plots enable a total view on the genome which may reveal a clustering of genes according to groups of nearby points. These clusters can for instance provide evidence for horizontal gene transfer according to groups of putative alien genes [1,2] or for translational selection according to groups of highly expressed genes [3,4].

As a standard method for scatter plot visualization of codon usage data, researchers mostly resort to the so-called correspondence analysis (CA) which has originally been developed for the analysis of contingency tables [5]. From the original formulation it is not completely clear how CA applies to codon counts. Because different preprocessing and normalization schemes have been proposed, the use of CA in codon usage studies has not been without controversy [6]. Nevertheless, CA has been applied for the analysis of many bacterial genomes, including those of *Escherichia coli *[1,3], *Bacillus subtilis *[4,7,8], *Borrelia burgdorferi *[9,10], *Chlamydia trachomatis *[11], *Mycoplasma genitalium *[12], *Helicobacter pylori *[13] and *Pseudomonas aeruginosa *[14].

Recently, self-organizing maps [15] have been proposed as an alternative visualization method for codon usage data [2,16,17]. Although this method provides a simultaneous clustering of the data which may be useful in certain contexts, it requires to choose the size of a discrete grid on which the genes are mapped in a non-linear way. The grid-size is a critical parameter of the method and directly controls the final clustering in the visualization. Unfortunately, the grid-size of self-organizing maps is a so-called *hyperparameter *which usually cannot be inferred from the data in an unsupervised manner. Therefore the resulting visualizations bare the risk of being highly subjective.

Here we present a visualization method, which has been tailored to the analysis of codon usage data while not depending on difficult to tune hyperparameters. Our visualization method is based on multidimensional scaling and a new similarity measure for codon usage data. In the following we first introduce our probabilistic similarity measure for codon usage tables and outline the corresponding algorithm for multidimensional scaling based on P-values. Then we provide some visualizations for the analysis of four microbial genomes and discuss our results in comparison with the results obtained from the classical correspondence analysis method.

## P-values for multidimensional scaling

For the analysis of codon usage tables we developed a special similarity measure which has been derived from the well-known chi-square test for the comparison of two distributions. Unlike the classical chi-square test we do not decide whether two distributions are equal or not, but instead we only use the corresponding P-values to compute a similarity measure for the underlying codon usage tables. For each pair of genes we compare the corresponding codon distributions on the basis of the codon frequencies in the two genes. For a suitable similarity score we average the P-values of the amino acid specific chi-square tests. We start with the counts  for codon  of amino acid *a*_*i *_in the *j*-th gene. These counts sum up to  over the number *L*_*i *_of different codons for amino acid *a*_*i*_. Note that *n*_*ij *_corresponds to the number of occurrences of amino acid *a*_*i *_in gene *j*. With these counts we compute the chi-square statistic for each pair (*j*, *k*) of genes:



The classical chi-square test for comparison of two distributions is based on the following proposition: under the null hypothesis that the corresponding samples were drawn from the same probability distribution, the variable  is asymptotically chi-square distributed with *L*_*i *_degrees of freedom. Here we do not perform a chi-square test, but rather calculate the P-value *P*_*ijk *_associated with the chi-square statistic . The P-values are obtained from the chi-square probability function which is an incomplete gamma function [18]. A small value of *P*_*ijk *_indicates a significant difference between the codon distributions of gene *j *and *k *with respect to amino acid *a*_*i*_. For a number of *M *genes in a genome we then assemble the *M *× *M *matrix **S **of similarity scores with non-negative elements



where *n*_*a *_is the number of amino acids. Note that **S **has unit diagonal elements, i.e. *S*_*jj *_= 1, because the P-value for tables with identical counts is one. Consequently all off-diagonal elements are in the range [0, 1].

In order to derive a suitable low-dimensional point representation of genes we apply classical multidimensional scaling (see e.g. [19]) to the above similarities. The objective is to find a two-dimensional point configuration with interpoint distances reflecting the codon usage similarities of the corresponding genes. To perform classical scaling based on similarities we first transform the similarity matrix **S **into a positive semi-definite matrix **C **by subtracting the smallest eigenvalue λ_min _of **S **from all of its diagonal elements:

**C **= **S **- λ_min_**I **    (3)

where **I **is the *M *× *M *identity matrix. Note that this transformation preserves the equality of diagonal elements. With the *M *× *M *centering matrix **H **with elements



we finally obtain the matrix

**B **= **HCH**.     (5)

It can be shown that for a positive semi-definite matrix **C **the distance matrix **D **with elements obtained by the standard transformation  is Euclidean and **B **is a centered inner product matrix ([19], pp. 402). Therefore principal components can be obtained from (partial) eigenvalue decomposition of **B**. Thus, for 2D-visualization we compute the two leading eigenvectors x_1 _and x_2 _of **B **associated with the largest and second largest eigenvalue, respectively. The *M *components of x_1 _and x_2 _provide the *x*_1 _and *x*_2 _coordinates for the *M *genes, which are utilized for scatter plot visualization.

## Experimental results

### Data sets

To evaluate our multidimensional scaling (MDS) approach, we focused on visualizations of ribosomal protein genes and putative alien genes for different microbial genomes. Ribosomal protein genes belong to the class of highly expressed genes which tend to use codons associated with the prevalent tRNAs present in the organism. If translational selection is one of the main sources for codon preferences in a particular genome, then codon usage can in turn be used for the prediction of putative highly expressed genes [20]. Another source of codon usage variation in microbial genomes is provided by the insertion of foreign DNA by means of horizontal gene transfer. Thus, putative alien genes may also be predicted on the basis of codon usage analysis [2,21]. While ribosomal protein genes can be identified from the annotations of completely sequenced genomes, reliable information about putative alien genes is much more difficult to obtain. We combined predictions of the SIGI-HMM tool [22] with existing references from the literature in order to obtain suitable test sets for our evaluations. SIGI-HMM is based on a Hidden Markov Model for the detection of genomic islands and, in contrast to our MDS-based visualization method, it explicitly uses information about the locations of genes on the corresponding chromosomes. However, unlike MDS, SIGI-HMM does not consider codon usage correlations between different amino acids. Using the two complementary kinds of information exclusively, both methods provide completely different approaches to codon usage analysis [22].

For the evaluation of the MDS-based visualizations we analyzed the microbial genomes of *Escherichia coli K-12*, *Bacillus subtilis*, *Vibrio cholerae *and *Thermus thermophilus HB8*. We used annotated DNA sequence data in the EMBL format publicly available from EBI [23]. Ribosomal protein genes were extracted from the datasets of the completely annotated genomes. Putative alien genes were selected according to the following information: On chromosome 1 of *V. cholerae *two genomic islands were predicted by SIGI-HMM that comprise a gene cluster for a toxin-coregulated pilus and fragments of a temperate filamentous phage described in [24]. Both clusters are closely associated with the pathogenicity of *V. cholerae*. For *Bacillus subtilis *10 integrated prophages have been described based on experimental evidence and theoretical considerations [25-28]. Nine of these prophages overlap with genomic islands as predicted by SIGI-HMM. For *Escherichia coli *K-12 the authors of [29] used different compositional variables and estimated that about 18% of the genome have been imported by horizontal gene transfer. In contrast, SIGI-HMM predicted 580 genes (13,6%) to be putatively alien. The largest genomic islands comprise the cryptic prophages CP4-6, DLP12, e14, Rac, Qin, CP4-44, CPS-53, Eut, CP4-57, and the phage-like element KpLE2 (reviewed in [30]). For the extremophilic bacterium *Thermus thermophilus HB8 *no genomic islands have been described so far. SIGI-HMM predicted a contiguous gene cluster of 5 genes associated with functions in cell wall biosynthesis to be putative alien. The total number of putative alien genes and the number of ribosomal protein genes for all species considered here are summarized in table 1. Additional file 1 provides a detailed list of all putative alien genes used for the visualization.

**Table 1:** Number of genes used for the visualization for all species under consideration. Given are the number of putative alien genes, the number of ribosomal protein genes and the total number of genes on the respective chromosomes.

species	# genes (total)	# ribosomal protein genes	# putative alien genes
*E. coli*	4254	61	206
*B. subtilis*	4106	57	317
*V. cholerae *Chr1	2736	64	41
*V. cholerae *Chr2	1092	0	216
*T. thermophilus*	1973	60	5

### Visualization

We compared our multidimensional scaling (MDS) approach with the correspondence analysis (CA) method as implemented in the *CodonW *program [31] of J. Peden. Computations were based on *relative synonymous codon usage *(*RSCU*) values which is the most common way to perform CA on codon usage data [6]. For both methods the resulting coordinates were normalized according to a unit variance of the leading two factors and principal components, respectively.

**Figure 1:**
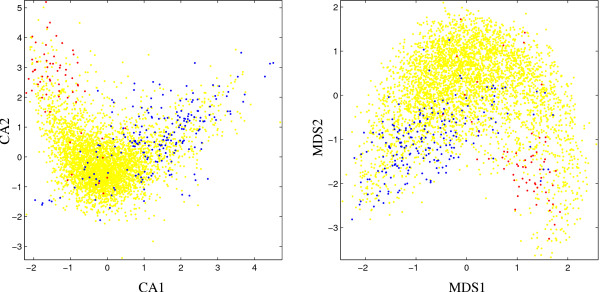
Scatter plots for *E. coli *based on first two components of correspondence analysis (left, CA) and P-value based multidimensional scaling (right, MDS), respectively. Red dots: ribosomal protein genes; blue dots: putative alien genes; yellow dots: all other genes.

The CA-based visualization for E. coli (Fig. 1) shows the typical "rabbit head" structure which has been described in [1]. The "ears" correspond to two branches of the distribution with low density. The "left ear" in the upper left corner shows a cluster of ribosomal protein genes while putative alien genes are mainly located around the other branch of the distribution. The MDS plot in Fig. 1 shows a similar picture with ribosomal protein genes and putative alien genes again concentrated in the two branches of the distribution which here appears rotated by 180 degrees. Comparing the visualizations, most of the ribosomal protein genes are well-clustered in both plots while putative alien genes are slightly more concentrated in the MDS plot. Note that the CA-based visualization shows an outlier at the lower boundary of the plot which is not among the putative alien genes.

**Figure 2:**
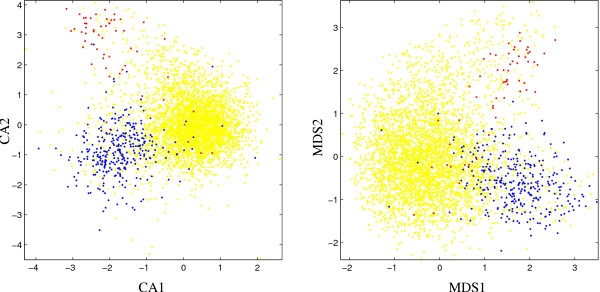
Scatter plots for *B. subtilis *based on first two components of correspondence analysis (left, CA) and P-value based multidimensional scaling (right, MDS), respectively. Red dots: ribosomal protein genes; blue dots: putative alien genes; yellow dots: all other genes.

For *B. subtilis *(Fig. 2) both visualization methods show a good clustering of putative alien genes and ribosomal protein genes in the branches of the distribution. Again the lower boundary of the CA plot is determined by an outlier which does not belong to the set of putative alien genes.

**Figure 3:**
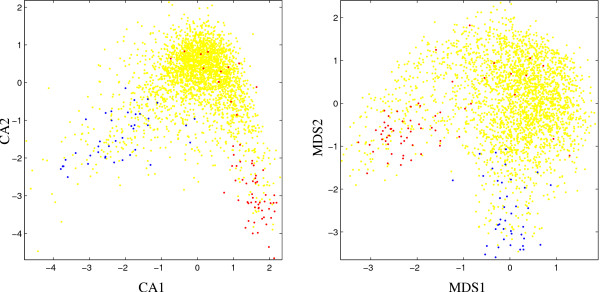
Scatter plots for *V. cholerae *(chromosome 1) based on first two components of correspondence analysis (left, CA) and P-value based multidimensional scaling (right, MDS), respectively. Red dots: ribosomal protein genes; blue dots: putative alien genes; yellow dots: all other genes.

**Figure 4:**
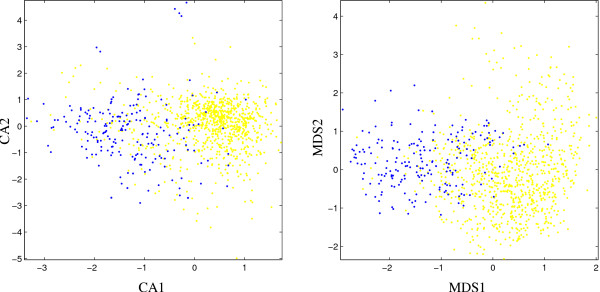
Scatter plots for *V. cholerae *(chromosome 2) based on first two components of correspondence analysis (left, CA) and P-value based multidimensional scaling (right, MDS), respectively. Red dots: ribosomal protein genes; blue dots: putative alien genes; yellow dots: all other genes.

For the first chromosome of *V. cholerae *(Fig. 3) the comparison shows a similar situation as for *B. subtilis*: in both plots, most of the ribosomal protein and putative alien genes are well-clustered in the two branches of the distribution. In the lower left corner of the CA-based plot there is an outlier which is not in the set of putative alien genes. As chromosome II of *V. cholerae *does not contain any ribosomal protein genes, the visualization of this replicon is restricted to putative alien genes (Fig. 4). These genes are slightly more concentrated in the MDS-based plot. Again, the lower boundary of the CA-plot is determined by an outlier which is not among putative alien genes.

**Figure 5:**
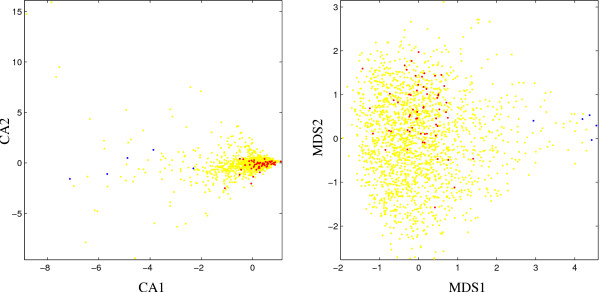
Scatter plots for *T. thermophilus *based on first two components of correspondence analysis (left, CA) and P-value based multidimensional scaling (right, MDS), respectively. Red dots: ribosomal protein genes; blue dots: putative alien genes; yellow dots: all other genes.

For *T. thermophilus *(Fig. 5) the outlier sensitivity of CA results in a highly distorted plot which makes it difficult to draw any conclusions from the visualization at all. While ribosomal protein genes are clumped together with the remaining genes in a small region of the plot, putative alien genes are widespread in a region of low density. In contrast, the MDS-based plot shows a specific proximity of putative alien genes in a tail at the right border and the ribosomal protein genes at least show some weak clustering in the upper right part of the core distribution.

## Conclusion

We proposed an approach for the visualization of codon usage data, using multidimensional scaling (MDS). In that context we introduced a novel similarity measure for codon usage tables, which has been derived from the classical chi-square test. An important feature of our P-value based similarity measure is that it does not involve any hyperparameters. Therefore a subjective "bias" on the visualization due to user-adjusted parameters is effectively avoided. Our comparisons with the widely-used correspondence analysis (CA) method in most cases showed a slightly better clustering of putative alien genes for our P-value based visualization. In particular the results indicate that our approach is more robust than the CA-based visualization method. The outlier-sensitivity of CA becomes apparent in the plots for all species considered here and has already been observed in previous studies [9]. While in most cases the CA-based visualizations are still useful in terms of a suitable clustering of ribosomal protein and putative alien genes, for *T. thermophilus *that sensitivity results in an inappropriate plot which complicates interpretation.

## Supplementary Material

Additional File 1 provides an Excel table (XLS) containing a detailed list of all putative alien genes used for the visualization. Click here for file
